# Cytokeratin 5/6 expression in bladder cancer: association with clinicopathologic parameters and prognosis

**DOI:** 10.1186/s13104-018-3319-4

**Published:** 2018-03-27

**Authors:** Atif Ali Hashmi, Zubaida Fida Hussain, Muhammad Irfan, Muhammad Muzzammil Edhi, Sarah Kanwal, Naveen Faridi, Amir Khan

**Affiliations:** 1 Liaquat National Hospital and Medical College, Karachi, Pakistan; 2 Brown University, Providence, RI USA; 3 Kandahar University, Kandahar, Afghanistan

**Keywords:** Urothelial carcinoma, Bladder cancer, Cytokeratin 5/6, CK5/6, Deep muscle invasion

## Abstract

**Objectives:**

Well differentiated keratinized squamous component as a part of urothelial carcinoma can be easily appreciated; however non-keratinizing squamous differentiation closely resembles urothelial differentiation. In addition prognostic significance of CK 5/6 expression in the absence of apparent squamous differentiation is still unclear. Therefore, in the present study we aimed to evaluate the frequency of CK 5/6 expression in 127 cases of urothelial carcinoma and its prognostic significance in loco-regional population.

**Results:**

Positive CK5/6 expression was noted in 6.3% (8 cases) and 13.4% (17 cases) revealed focal positive CK 5/6 expression. On the other hand, 80.3% (102 cases) showed negative CK5/6 staining. Significant association of CK5/6 expression was noted with tumor grade and muscularis propria invasion, however no significant association was noted with overall and disease free survival. On the basis of the results of our study we can conclude that CK5/6 is an independent prognostic biomarker in urothelial carcinoma and therefore can be used in the prognostic stratification of the patients with bladder cancer.

## Introduction

Bladder cancer is among one of the most common malignancy in males worldwide and its incidence is even higher in developing countries owing to certain endemic infections [1, 2]. Urothelial carcinoma is the most common histologic subtype of bladder cancer, while Squamous cell carcinoma is seen in association with bladder stones and schistosomiasis. Muscle invasion is one of the most important prognostic factors in bladder cancer; as it necessitates radical therapy and poor 5 year disease free survival [3, 4]. While, well differentiated keratinized squamous component as part of urothelial carcinoma can be easily appreciated; non-keratinizing squamous differentiation closely resembles urothelial differentiation. On the other hand, WHO/ISUP don’t recommend routine use of immunohistochemical markers to identify squamous differentiation in urothelial carcinoma. Conversely, markers of squamous differentiation like p63, p40, CK5/6 can be positive in urothelial carcinoma [5]. In addition prognostic significance of CK 5/6 expression in the absence of apparent squamous differentiation is still unclear. Therefore, in the present study we aimed to evaluate the frequency of CK 5/6 expression in urothelial carcinoma and its prognostic significance in loco-regional population.

## Main text

Total 240 diagnosed cases of urothelial carcinoma specimens were selected from records of pathology department. All patients underwent surgeries at Liaquat National hospital, Karachi from January 2010 till December 2014 over a period of 5 years. The study was approved by research and ethical review committee of Liaquat National Hospital and informed written consent was taken from all patients at the time of surgery. Hematoxylin and eosin stained slides and paraffin blocks of all cases were retrieved and new sections were cut when necessary. Slides of all cases were reviewed by two senior histopathologists and pathologic characteristics like histologic type, tumor grade, lamina propria invasion, muscularis propria invasion were evaluated. Clinical records of 61 patients were available and are thus reviewed from institutional records to evaluate history of radiation and chemotherapy and recurrence status. Moreover, representative tissue blocks of 127 cases were available for CK5/6 immunohistochemistry.

**Fig. 1:**
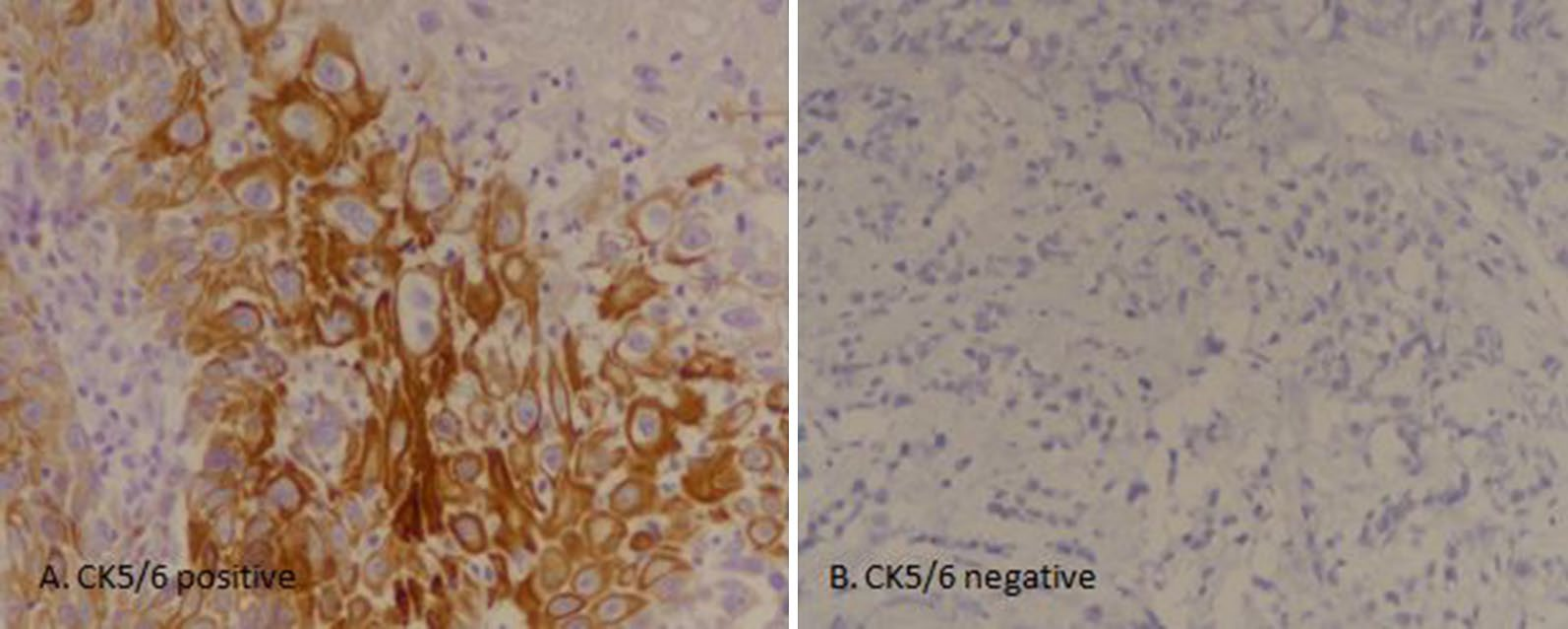
CK5/6 expression in bladder carcinoma

CK5/6 IHC was performed by using FLEX Monoclonal Mouse Anti-human Cytokeratin 5/6, clone D5/16 B4 (Lot No. 20042129) by DAKO envision method according to manufacturers protocol on 127 cases of urothelial carcinoma (on representative tissue blocks). Results of IHC staining were interpreted by two senior histopathologists with more than 5 years experience of reporting histopathology and immunohistochemistry and they were blinded by other histopathological features of the tumors. For quantification, at least 1000 cells were counted in 10 HPFs (40×). Intermediate to strong cytoplasmic and membranous staining in more than 10% of tumor cells was considered positive. Weak to intermediate staining in < 10% was taken as focal positive, while no staining was considered as negative (Fig. 1).

Recurrence status and follow-up were evaluated by reviewing hospital medical records. Overall survival was taken as time from surgical excision till death or last follow-up and disease free survival was defined as time between surgical excision and local recurrence or distant metastasis, death or last follow-up.

All cases of primary urothelial carcinoma were included in the study. Cases of squamous cell carcinoma or those cases of urothelial carcinoma showing divergent differentiation (including squamous differentiation) were excluded from the study.

Statistical package for social sciences (SPSS 21) was used for data compilation and analysis. Mean and standard deviation were calculated for quantitative variables. Frequency and percentage were calculated for qualitative variables. Chi square was applied to determine association. Student t test or Mann Witney test were applied to compare difference in means among groups. Survival curves were plotted using Kaplan–Meier method and the significance of difference between survival curves were determined using log-rank ratio. *P* value ≤ 0.05 was taken as significant.

Mean age of patients was 63.23 + 13.9 years with male to female ratio of 3:1. 95.8% specimens were of transurethral resections. 50.8% (122 cases) were of high grade morphology, whereas 49.2% (118 cases) showed low grade histology. Lamina propria invasion was seen in 30.4% (73 cases), while muscularis propria invasion was noted in 22.9% (55 cases). Mean follow up of patients involved in the study was 22.0 + 13.74 months and recurrence was seen in 45.9% (28 cases) as presented in Table 1.

**Table 1:** Clinicopathologic features of bladder carcinoma

	n (%)
Age (years)^a^	63.28 ± 13.90
Follow up (months)^a^	22.00 ± 13.74
Gender	
Male	182 (75.8)
Female	58 (24.2)
Tissue type	
Transurethral resection	230 (95.8)
Radical cystectomy	10 (4.2)
Tumor grade	
Low grade papillary urothelial carcinoma	118 (49.2)
High grade papillary urothelial carcinoma	122 (50.8)
Lamina propria invasion	
Present	73 (30.4)
Absent	167 (69.6)
Deep muscle invasion	
Present	55 (22.9)
Absent	140 (58.3)
Can’t assessed	45 (18.8)
Recurrence (n = 61)	
Yes	28 (45.9)
No	33 (54.1)
Survival status (n = 61)	
Alive	52 (85.2)
Expired	9 (14.8)

^a^Mean ± SD

**Fig. 2:**
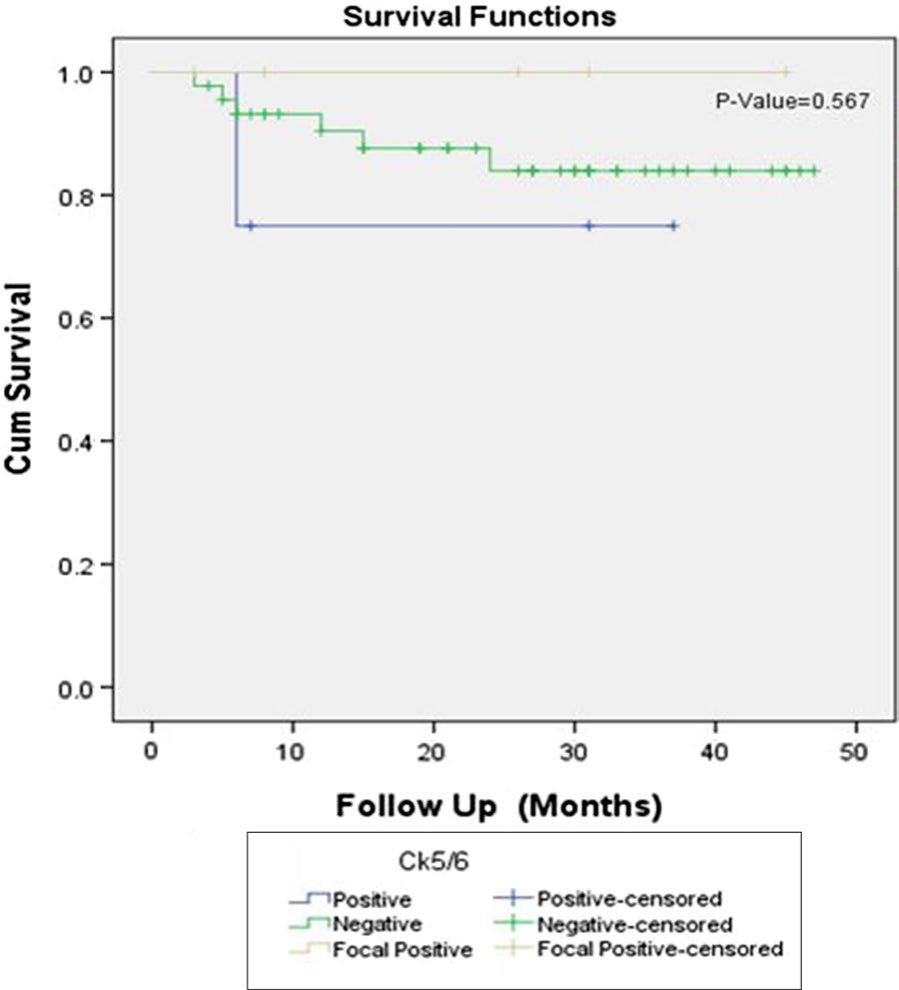
Kalpien–Meier for CK5/6 overexpression in bladder cancer for overall survival

**Fig. 3:**
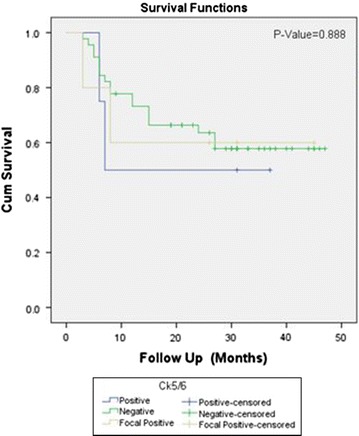
Kalpien–Meier for CK5/6 overexpression in bladder cancer for recurrence

Positive CK5/6 expression was noted in 6.3% (8 cases) and 13.4% (17 cases) revealed focal positive CK 5/6 expression. On the other hand, 80.3% (102 cases) showed negative CK5/6 staining. Significant association of CK5/6 expression was noted with tumor grade and muscularis propria invasion, however no significant association was noted with lamina propria invasion and disease free survival (Table 2 and Figs. 2 and 3).

**Table 2:** Association of CK 5/6 expression with clinicopathologic features of bladder cancer

	CK 5/6 n (%)				P value
	Positive	Negative	Focal positive	Total	
Gender					
Male	8 (100)	77 (75.5)	8 (47.1)	93 (73.2)	0.014
Female	0 (0)	25 (24.5)	9 (52.9)	34 (26.8)	
Total	8	102	17	127	
Age group					
≤ 25 years	0 (0)	1 (1)	0 (0)	1 (0.8)	0.613
26–50 years	2 (25)	26 (25.5)	2 (11.8)	30 (23.6)	
> 50 years	6 (75)	75 (73.5)	15 (88.2)	96 (75.6)	
Total	8	102	17	127	
Tissue type					
Transurethral resection	6 (75)	99 (97.1)	17 (100)	122 (96.1)	0.049
Radical cystectomy	2 (25)	3 (2.9)	0 (0)	5 (3.9)	
Total	8	102	17	127	
Tumor grade					
Low grade	0 (0)	59 (57.8)	5 (29.4)	64 (50.4)	0.000
High grade	8 (100)	43 (42.2)	12 (70.6)	63 (49.6)	
Total	8	102	17	127	
Lamina propria invasion					
Present	5 (62.5)	31 (30.4)	3 (17.6)	39 (30.7)	0.081
Absent	3 (37.5)	71 (69.6)	14 (82.4)	88 (69.3)	
Total	8	102	17	127	
Deep muscle invasion					
Present	5 (62.5)	19 (18.6)	2 (11.8)	26 (20.5)	0.049
Absent	1 (12.5)	47 (46.1)	10 (58.8)	58 (45.7)	
Can’t assessed	2 (25)	36 (35.3)	5 (29.4)	43 (33.9)	
Total	8	102	17	127	
Recurrence (n = 54)					
Yes	2 (50)	18 (40)	2 (40)	22 (40.7)	1.000
No	2 (50)	27 (60)	3 (60)	32 (59.3)	
Total	4	45	5	54	
Survival status (n = 54)					
Alive	3 (75)	39 (86.7)	5 (100)	47 (87)	0.514
Expired	1 (25)	6 (13.3)	0 (0)	7 (13)	
Total	4	45	5	54	

Fisher exact test applied

P ≤ 0.05, considered as significant

In the present study we found that CK5/6 expression is low in urothelial carcinoma in our set up; however, its positivity signifies adverse prognostic features like higher tumor grade and muscularis propria invasion.

CK5/6 is a basal cytokeratin which normally expresses in squamous epithelium and in squamous cell carcinoma. Although diagnosis of squamous cell carcinoma in bladder is restricted to those tumors which show pure squamous differentiation in the absence of any urothelial component. Conversely, advanced urothelial carcinoma can show divergent differentiation (including squamous component) in up to 50% of cases and is associated with poor disease progression [6]. Morphologic diagnosis of squamous differentiation in urothelial carcinoma is based on the presence of either intercellular bridges or presence of keratinization in the form of keratin pearls or individual cell keratinization; however non-keratinizing or poorly differentiated squamous component can closely resemble urothelial carcinoma and therefore can’t be readily apparent. Gaisa et al. [7] performed IHC markers of squamous differentiation including CK5/6 and CK4/14; and found squamous differentiation in a high proportion of urothelial carcinoma without morphologic evidence of squamous differentiation. Langer et al. evaluated the prognostic value of keratin subtyping in urothelial carcinoma and revealed the prognostic impact of various cytokeratin staining in urothelial carcinoma including CK5/6.

## Limitations

One of the major limitations of our study was that we performed only single biomarker of squamous differentiation in our study; use of multiple markers like CK5/14 and CK4/14 could increase the sensitivity of the study. However, on the basis of the results of our study we can conclude that CK5/6 is an independent prognostic biomarker in urothelial carcinoma and therefore can be used in the prognostic stratification of the patients with bladder cancer.

## Abbreviations

IHC: immunohistochemistry
WHO: World Health Organization
ISUP: International Society of Urological Pathology
